# How many future dementia cases would be missed by a high‐risk screening program? A retrospective cohort study in a population‐based cohort

**DOI:** 10.1002/alz.14113

**Published:** 2024-07-18

**Authors:** Sebastian Walsh, Lindsay Wallace, Richard Merrick, Shabina Hayat, Robert Luben, Oliver Mytton, Louise Lafortune, Carol Brayne

**Affiliations:** ^1^ Cambridge Public Health University of Cambridge, Robinson Way Cambridge UK; ^2^ Department of Behavioural Science and Health University College London (UCL) London UK; ^3^ NIHR Biomedical Research Centre Moorfields Eye Hospital NHS Foundation Trust and UCL Institute of Ophthalmology London UK; ^4^ MRC Epidemiology Unit University of Cambridge Cambridge UK; ^5^ Great Ormond Street Institute of Child Health University College London London UK

**Keywords:** dementia, population health, prevention, risk prediction, risk reduction

## Abstract

**INTRODUCTION:**

Risk prediction models aim to identify those at high risk to receive targeted interventions. We aimed to identify the proportion of future dementia cases that would be missed by a high‐risk screening program.

**METHODS:**

We identified validated dementia risk prediction models from systematic reviews. We applied these to European Prospective Investigation of Cancer Norfolk, a large population‐based cohort of 30,387 individuals with 29 years of linked healthcare data.

**RESULTS:**

A maximum of 16.0% (14.7,17.2) and 31.9% (30.2,33.5) of cases arose from the highest risk decile and quintiles, respectively. For every 1000 people considered to be at high risk, a maximum of 235 (215, 255) developed dementia.

**DISCUSSION:**

Seven in every 10 cases of dementia arose from people at normal risk, and eight in every 10 people at high risk did not develop dementia. Individual‐level prevention approaches targeted at high‐risk groups are unlikely to produce large reductions in disease incidence at the population level.

**Highlights:**

- Dementia, a significant public health challenge, is not an inevitability of aging; risk reduction is possible.
- Several dementia risk prediction models have been validated in the general population, and these aim to identify people at high risk of the disease who can then be targeted with primary prevention interventions. An alternative prevention approach is to focus on interventions that reduce risk across the population, irrespective of risk status.
- In our study, seven out of every ten people who developed dementia during 29 year follow‐up were classed as ‘normal‐risk’ (rather than ‘high risk’) at baseline. Eight out of every ten people who were at high risk at baseline did not go on to develop dementia.
- Even if effective, dementia risk reduction efforts based upon targeted high‐risk approaches are unlikely to reduce incidence of disease at the population level.

## 1 BACKGROUND

It is estimated that there are around a million people with dementia in the UK,^1^ with an annual societal cost in England of over £31 billion (almost US$40 billion).^2^ A combination of a lack of clinically meaningful treatment options^3^ and evidence that incidence has declined^4^ has focused attention on strategies to reduce dementia risk. Observational studies have identified risk and protective factors for dementia,^5^ some of which are modifiable and could therefore be targets for public health policy action to reduce dementia risk in the population.^6^

Reducing disease by targeting risk factors is known as primary prevention,^7^ and two broad approaches have been described.^8^ The high‐risk individual approach, which has dominated dementia prevention research efforts to date,^9^ identifies those with higher levels of risk and targets these groups with prevention interventions to lower their risk. To this end, significant effort has been devoted to the development of dementia risk prediction models.^10^, ^11^, ^12^, ^13^ The alternative, the population‐level approach utilizes policy changes to reduce risk for everyone across the population (ie, irrespective of risk status). An individual‐level approach might include referring individuals with diabetes and who are physically inactive to a weight management program, whereas a population‐level approach would make investments in making a local area more amenable to active travel and leisure‐time exercise for all.^6^, ^14^

Disease risk can be considered as a continuum in the population. At the extreme end of the continuum are those with the greatest relative risk of disease (the group that the risk scores aim to identify and treat). However, because the vast majority of people lie somewhere in the middle of the continuum, at normal (ie, not high) risk, by sheer weight of numbers this is the group from which most incidence is hypothesized to arise in the population. Therefore, approaches targeting high‐risk individuals (even if effective) are expected to have little effect on overall disease occurrence; this is known as the prevention paradox.^8^ Despite this, high‐risk individual‐level approaches are often favored because policymakers want to target limited resources at the most at‐risk groups and because they consider population‐level interventions to be more complex and more “nanny state” and to require longer‐term cohesive policy environments.^15^ Evidence to guide the balancing of individual‐ and population‐level interventions for dementia risk reduction is therefore of paramount importance.

To our knowledge, the prevention paradox has never been empirically tested for dementia in a cohort that is population‐representative. If it holds, then any hypothetical screening program that aimed to identify individuals at high risk of dementia would in fact miss the majority of future cases, and therefore any intervention in this group (however effective) would have limited effect on reducing the future prevalence of dementia despite major investments. In this study, we used 29‐year follow‐up data from a population‐based cohort in England with linked healthcare data to empirically test the prevention paradox for dementia, using a variety of dementia risk prediction tools.

## 2 METHODS

### 2.1 Identifying dementia risk prediction models

We reviewed recent systematic reviews of dementia risk prediction models^10^, ^11^, ^12^, ^13^ to identify risk prediction models that have been internally and externally validated in general population cohorts. We included the best performing models that were designed to inform primary, rather than secondary, prevention (ie, we excluded models that included participants with early symptoms of cognitive impairment). Only models validated in cohorts with at least 5 years of follow‐up were included in order to allow sufficient time for a hypothetical primary prevention intervention program to take effect. Models with any data that could be feasibly collected at scale (eg, questionnaire‐based, blood samples, genetic data) in a hypothetical screening program were included.

### 2.2 Application to a UK population‐based sample

We applied each of the identified risk prediction models to the European Prospective Investigation of Cancer Norfolk (EPIC‐Norfolk) cohort. EPIC‐Norfolk recruitment and procedures have been described fully elsewhere.^16^ EPIC‐Norfolk recruited participants, commencing in 1993, aged between 40 and 79 (mean age 59.2) from the county of Norfolk, UK. A total of 30,445 were recruited, of whom 30,411 completed baseline questionnaire data, and 25,639 attended the baseline health check. The cohort was comparable to those recruited at the time for the Health Survey for England, except for a lower smoking profile.^16^ Data on dementia incidence for EPIC‐Norfolk participants were available through linkages to mortality data up to March 2022, secondary care data via Hospital Episode Statistics (HES) up to March 2022, and Mental Health Trust data (the health organizations where memory clinics are situated) until March 2019. All‐cause dementia and dementia subtype were defined using the International Classification of Disease codes as per Hayat et al.^17^

**RESEARCH IN CONTEXT**

1. **Systematic review**: We reviewed four recent systematic reviews of dementia risk prediction models. We identified five primary prevention models that were internally and externally validated for use in the general population. We applied each of these risk prediction models to the European Prospective Investigation of Cancer Norfolk population‐based cohort, which has 29 years of linked healthcare follow‐up data, to see what proportion of future dementia cases would have been classified as high risk at baseline and what proportion of cases arose from the normal‐risk (ie, not high‐risk) group.
2. **Interpretation**: Seven out of every 10 people who developed dementia were classified as normal risk (rather than high risk) at baseline. Moreover, eight out of every 10 people who were classified as high risk at baseline did not go on to develop dementia during the follow‐up. Dementia risk reduction approaches based upon targeting high‐risk groups are therefore unlikely to result in large reductions in disease incidence at the population level, even if successful.
3. **Future directions**: Future studies assessing this same research question could look to replicate these findings in other population‐based cohorts and investigate the effect of multiple screening rounds where data allow this. If blood‐based biomarkers are validated in the general population, the added value of incorporating them into risk prediction tools could also be analyzed. Population‐level approaches that aim to reduce everyone's risk, not just those categorized as high risk, should be considered.

Risk scores were applied as consistently as possible according to the originally derived risk scores. If available variables in EPIC‐Norfolk were not well matched to those in the original derivation cohorts, then risk score study authors were contacted for advice, and the appropriate course of action agreed between co‐authors (see Table S1 for full details of coding approach). We generated cohort‐derived high‐risk cut points of the top decile and quintile for each risk score, on the basis that these might produce feasible numbers of individuals to be referred to high‐risk intervention programs. Additionally, previously suggested cut points were identified from systematic reviews,^10^, ^11^, ^12^, ^13^ communication with risk score study authors, or other relevant literature. Due to the discrete nature of the risk scores, it was not possible to derive cut points that corresponded to exact proportions of the cohort, so the cut points corresponding to the next smallest percentage were used and reported alongside the results.

We derived the risk scores and cut points in the whole EPIC‐Norfolk sample using the entire baseline data. We then performed a retrospective cohort analysis, restricted to incident cases of dementia occurring at least 5 years after baseline. We quantified the proportion of the incident dementia cases arising from the high‐risk group, for each risk score and cut point. This represented the maximum proportion of future cases that the hypothetical screening program (with no recall rounds) could have identified. We investigated whether cases arising from the high‐risk groups were diagnosed earlier than other cases and the relationship between baseline risk score and mortality. We performed subgroup analyses to examine risk score performance in specific age groups (eg, those aged 50 to 59 years at baseline), and for different dementia subtypes (Alzheimer's disease, vascular dementia, other dementias).

### 2.3 Sensitivity analyses

In the primary analysis, those with missing data were excluded from the analysis (complete case analysis). We compared the age, sex, socioeconomic status (using Townsend index^18^), and incident dementia status between those included and those excluded in the complete case analysis. Additionally, we performed two sensitivity analyses to test the effect of missing data on our findings. (1) We removed from the risk score variables that were outliers (eg, by an order of magnitude) for the amount of missing data. (2) We included all individuals, including those with missing data, by creating percentage (rather than absolute) risk scores using whatever data were available for each individual. We also performed sensitivity analyses excluding incident cases in the first 10 years of follow‐up.

Some risk scores included variables related to cognitive and social activity, for which equivalent data were not available in EPIC‐Norfolk, and these variables were therefore excluded from the risk scores in our primary analysis. However, occupational social class and marital status were available, which we considered in sensitivity analyses as potential proxy variables for cognitive and social activity, respectively (see Table S1 for coding approach).

### 2.4 Statistical analysis

Confidence intervals (CIs) for proportions were calculated using the formula ± 1.96 * √(*p*(1−*p*)/*n*), where *p* represents the sample proportion and *n* the sample size.

Differences in age at diagnosis between cases arising from high‐risk and normal‐risk groups were tested using linear regression models. All models were adjusted for baseline age as the high‐risk groups were older at baseline than the normal‐risk groups, with the exception of apolipoprotein ε4 (APOE ε4) carriership (this model was unadjusted). All analyses were completed using Stata 17.0.

### 2.5 Ethics and patient and public involvement

The EPIC‐Norfolk study was approved by the Norfolk Local Research Ethics Committee (REC Ref. 98CN01). EPIC‐Norfolk has permission to follow up with participants via medical record linkage through Section 251, Application No. 059, and explicit participant consent. Data release for this study was approved by the EPIC‐Norfolk team.

EPIC‐Norfolk has been supported by a participant advisory panel since 2010, which includes decisions on the prioritization of research conducted in the study, and provides a participant perspective on the dissemination of findings. A draft of the proposal for our specific study was also presented to the University of Hertfordshire's Public Involvement in Research Group, which provided feedback that informed the study design and presentation of results to maximize the impact and clarity of message.

## 3 RESULTS

### 3.1 Risk prediction models identified from systematic reviews

We identified five relevant risk prediction models from the existing systematic reviews, and these are detailed in Table 1. The mean age in the cohorts used to develop the models ranged from 50 years for the Cardiovascular Risk Factors, Aging, and Dementia (CAIDE) score^19^ to the mid‐60s for the Lifestyle for Brain Health (LIBRA)^20^ and Verhaaren et al.^21^ and the early‐70s for the validation of the Australian National University Alzheimer's Disease Risk Index (ANU–ADRI) score.^22^ Two versions of the CAIDE score were produced, with and without APOE ε4 carriership, incorporated alongside demographic and lifestyle variables.^19^ The LIBRA score is the only score based entirely on modifiable factors (whereas others include factors like age and sex), with the explicit intention to produce an index that informs participants of their “dementia prevention potential” rather than their dementia risk.^20^ Unlike the other risk scores, for which beta weights from regression models were transformed into component scores for a replicable risk index, Verhaaren et al. reported only the outputs from a logistic regression model with three variables: age, sex, and APOE ε4 carriership status.^21^ For this model, we therefore chose to consider a hypothetical screening program that used APOE ε4 carriership alone to indicate those at high risk, and we report age‐ and sex‐specific results. The coding approach for each risk score is described fully in Table S1.

**TABLE 1:** Characteristics of identified dementia risk scores to inform primary prevention.

Model	Derivation cohort	Accuracy (AUC, 95% CI)	Follow‐up time (years)	Baseline age (mean, SD)	Variables included	Outcome measure	External validation (Cohort, AUC)	High‐risk cut point(s)
**CAIDE 1** ^19^	CAIDE study, Finland	0.77 (0.71, 0.83)	20	50.4 (6.0)	Age, sex, education, systolic blood pressure, BMI, total cholesterol, physical activity	All‐cause dementia	Kaiser Permanente cohort, 0.75^32^	≥9/15^19^ ≥ 6/15^33^
**CAIDE 2** ^19^	CAIDE study, Finland	0.78 (0.72, 0.84)	20	50.4 (6.0)	CAIDE 1 + APOE ε4 status	All‐cause dementia		
**LIBRA** ^20^	Maastricht aging study, Netherlands	0.60 (0.53, 0.67)	12	65.0 (8.7)	CHD, diabetes, total cholesterol, hypertension, depression, obesity, smoking, physical inactivity, renal disease, alcohol use, diet, cognitive activity	All cause dementia	CAIDE cohort, 0.67^34^	Top tertile^20^ ≥ SD above mean^35^
**ANU‐ADRI** ^22^	Derived from meta‐analyses of observational studies, rather than in a specific population‐representative cohort				Age, sex, education, diabetes, TBI, depressive symptoms, smoking, social networks, cognitively activities, alcohol consumption, physical activity, fish intake, pesticide exposure	Alzheimer's disease (primarily), all cause also reported	Rush MAP, 0.73 KP, 0.64 CVHS, 0.73^22^ AGES‐Reykjavik, 0.74^13^	Top 25%^22^
**APOE ε4 status** ^21^	Rotterdam study, Netherlands	0.82 (0.80, 0.83)	10	66.2 (11.2)	Age, sex, and APOE ε4 carriership	Alzheimer's disease	AGES‐Reykjavik, 0.73	n/a

Abbreviations: AUC, area under the curve; AGES, age, gene/environment, susceptibility study; APOE ε4, apolipoprotein ε4; BMI, body mass index; CHD, coronary heart disease; CI, confidence interval; CVHS, cardiovascular health cognition study; KP, Kungsholmen project; Rush MAP, Rush Memory and Aging Project; SD, standard deviation; TBI, traumatic brain injury.

### 3.2 Performance of risk scores in EPIC‐Norfolk cohort

A total of 3801 (12.5%) people in the EPIC‐Norfolk cohort developed dementia, at least 5 years after baseline data collection, according to the linked healthcare records. Table 2 reports the proportion of cases that would have been considered at high dementia risk at baseline, under the different proposed cut points. Between 9.8% (95% CI 8.7, 10.8) (LIBRA) and 16.0% (95% CI 14.7, 17.2) (ANU–ADRI) of cases arose from those in the highest risk decile, while between 23.9% (95% CI 22.5, 25.3) (CAIDE 1) and 31.9% (30.2, 33.5) (ANU–ADRI) of cases arose from the highest risk quintile.

**TABLE 2:** Performance of risk prediction scores in EPIC‐Norfolk.

Risk score	High‐risk threshold	Cut‐point value	Percentage of cohort high risk at baseline, % (95% CI)	Percentage of cases arising from high‐risk group, % (95% CI)	Dementia cases/ 1000 high‐risk,^a^ *n* (95% CI)
**Cut‐offs by decile**					
CAIDE 1 (min 0, max 15)	Top 10%	≥11	8.6 (8.3, 8.6)	12.8 (11.8, 13.9)	186 (171, 201)
	Top 20%	≥10	16.3 (15.9, 16.7)	23.9 (22.5, 25.3)	183 (172, 194)
CAIDE 2 (min 0, max 19)	Top 10%	≥13	8.4 (8.0. 8.8)	15.7 (14.3, 17.1)	235 (215, 255)
	Top 20%	≥12	14.9 (14.4, 15.4)	27.0 (25.3, 28.7)	227 (212, 242)
LIBRA (min −2.7, max 11.7)	Top 10%	≥4.2	8.3 (7.9, 8.6)	9.8 (8.7, 10.8)	147 (132, 163)
	Top 20%	≥3.1	19.0 (18.5, 19.5)	24.2 (22.6, 25.8)	159 (148, 170)
ANU‐ADRI (min −11, max 66)	Top 10%	≥11	8.8 (8.4, 9.1)	16.0 (14.7, 17.2)	227 (209, 244)
	Top 20%	≥6	19.8 (19.2, 20.2)	31.9 (30.2, 33.5)	202 (191, 213)
APOE ε4 carriership	Male		28.3 (27.4, 29.2)	43.6 (40.7, 46.6)	172 (158, 186)
	Female		28.7 (27.8, 29.4)	44.3 (41.8, 46.7)	213 (199, 227)
**Cut‐offs from literature**					
CAIDE 1	FINGERS trial^19^	≥9	27.4 (26.8, 27.9)	39.5 (37.9, 41.1)	180 (172, 189)
	Derivation threshold^33^	≥6	68.5 (68.0, 69.0)	86.2 (85.0, 87.3)	157 (152, 162)
LIBRA	≥ Mean + 1 SD	≥3.3	15.4 (14.9, 15.8)	19.2 (17.8, 20.7)	156 (145, 168)
	Top tertile	≥2.1	31.2 (30.6, 31.8)	37.8 (36.0, 39.6)	151 (143, 160)
ANU‐ADRI	Validation threshold^22^	≥5	22.5 (21.9, 23.0)	35.0 (33.3, 36.7)	195 (185, 205)

Abbreviations: ANU‐ADRI, Australian National University–Alzheimer's disease risk index; APOE ε4, apolipoprotein ε4; CAIDE, cardiovascular risk factors, aging, and dementia; CI, confidence interval; LIBRA, lifestyle for brain health; SD, standard deviation.

^a^ Calculated as number of cases arising from high‐risk group for every 1000 people in high‐risk group (to aid comparison between risk scores).

For every 1000 people placed in the high‐risk group, between 147 (95% CI 132, 163) (LIBRA, top decile) and 235 (95% CI 215, 255) (CAIDE 2, top decile) people developed dementia during the 29‐year follow‐up (for reference, the 12.5% total incidence rate during follow‐up means 125/1000 would be expected to develop dementia if the risk scores had zero predictive ability).

Over 40% of dementia cases arose from APOE ε4 carriers. APOE ε4 status performed less well than the CAIDE (1 and 2) and ANU–ADRI risk scores when considering number of cases for every 1000 people considered at risk (Table 2). This may have been driven by the inclusion of age in these risk scores because in age‐stratified analyses (Table S2) APOE ε4 status performed comparably to or better than the other scores for the number of cases per 1000 at high risk.

**FIGURE 1:**
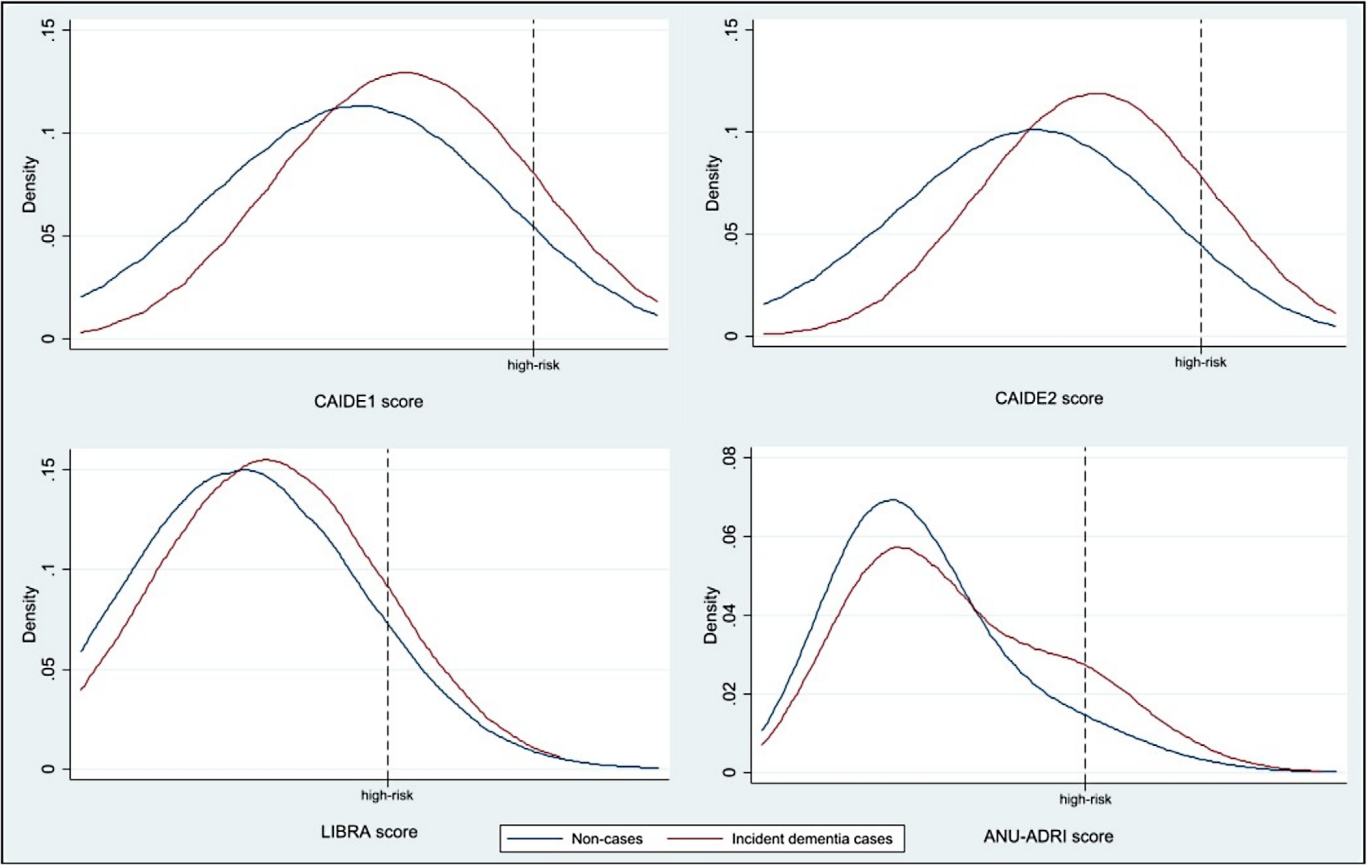
Kernel density plots showing distribution of baseline risk scores for those who remained dementia free (blue) and those who went on to develop dementia (red). The area to the right of the dotted black line indicates the highest decile of risk for each score. ANU‐ADRI, Australian National University–Alzheimer's disease risk index; CAIDE, cardiovascular risk factors, aging, and dementia; LIBRA, lifestyle for brain health.

Figure 1 presents the distributions of the risk scores by incident dementia status, showing that the curve for incident cases is to the right (indicating higher average risk scores) of the non‐cases but that the two curves are not sufficiently divergent for any single cut point to meaningfully segment the population into those that will or will not develop disease. The vast majority of cases will always arise from the group not deemed to be at high risk.

Cases arising from high‐risk groups were generally slightly younger at diagnosis than those from normal‐risk groups, after adjusting for their higher age at baseline (Table S3). Cases arising from the high‐risk group (top quintile) of CAIDE 2, LIBRA, and ANU–ADRI were 0.6, 0.6, and 1.6 years younger at diagnosis, respectively (there was no difference in diagnosis age for CAIDE 1). The average age at diagnosis for APOE ε4 carriers was 2.1 (men) and 2.6 (women) years younger than for non‐carriers. Higher risk scores were associated with increased mortality. Compared to the rest of the cohort, odds ratios for death for the top risk quintile (adjusted for baseline age) ranged from 1.43 (95% CI 1.32, 1.55) for CAIDE 1 to 2.26 (95% CI 2.04, 2.52) for ANU–ADRI.

Subgroup analyses considering the risk score performance in specific age groups (Table S2) and by diagnostic subtype (Table S4) showed a risk score performance similar to that of the primary analysis.

Those with missing data for each risk score were older (range 0.8 years older [CAIDE 2] to 2.6 years older [CAIDE 1]) and lived in neighborhoods that had slightly higher deprivation ranks (2.4% [APOE ε4] to 4.8% [CAIDE 1]) (Table S5). Women were slightly more likely to have missing data than men for all risk scores except LIBRA, in which the inverse was seen. There were no differences by dementia outcome status. Various sensitivity analyses testing the effect of missing data (Tables S6 and S7), excluding incident cases from the first 10 years of follow‐up (Table S8), and using proxy variables for cognitive and social engagement (Table S9) generated results similar to those of the primary analysis.^23^, ^24^, ^25^, ^26^

## 4 DISCUSSION

### 4.1 Main findings

In this broadly population‐representative sample with up to 29 years of follow‐up, around seven in every 10 cases of dementia arose from people categorized as normal risk (ie, not in the highest quintile of the risk scores at baseline) (Table 2). Moreover, for every 100 people who were considered eligible, around 80 would not go on to develop dementia (Table 2).

The distributions of baseline risk scores for people who were later diagnosed with dementia were 2% to 10% higher than those who were not diagnosed during follow‐up (Figure 1 and Table S7), and cases arising from the high‐risk group were slightly younger at diagnosis (Table S3). This indicates that the risk scores do possess some predictive capability for dementia risk. However, this discriminative capability was not enough to segment the population with sufficient accuracy. Given the large proportion of future cases that would be missed by a screening program aimed at high‐risk groups, the ability to reduce future dementia prevalence would be very limited.

Approximately 44% of cases occurred in those who carried at least one APOE ε4 allele (Table 2). In 50‐ to 59‐year‐olds, this was higher for both men (50.9% [95% CI 44.3%, 57.6%]) and women (57.2% [95% CI 50.7%, 63.7%]). While this far exceeds the proportions for any of the (predominantly lifestyle‐based) risk scores, almost a third (30%) of the total baseline cohort were APOE ε4 carriers, meaning that it would be challenging to implement a targeted intervention program for such a large group (and this would be in addition to the cost of genotyping the whole population in order to identify the group). Further, approximately 80% of APOE ε4 carriers did not develop dementia during the long follow‐up. CAIDE 2, which incorporates age and APOE ε4 status, and ANU–ADRI, which weights age very highly in its scoring (Table S1), generally performed better than CAIDE 1 and LIBRA, indicating that age and genetic status were likely stronger predictors of dementia risk than lifestyle factors. LIBRA performed the least well of all the risk scores. Yet, because LIBRA is the only score to include exclusively modifiable factors, these results probably represent the proportion of cases identified by the better performing scores that are actually amenable to a lifestyle‐based prevention intervention.

### 4.2 Findings in context

Geoffrey Rose postulated that, where disease risk can be considered continuously distributed in the population, most incident cases will arise from the large group in the middle of the risk distribution.^8^ Our analyses represent empirical evidence that Rose's hypothesis does apply to dementia. It logically follows that large reductions in dementia incidence cannot be achieved by only targeting those categorized as being at high risk. Instead, reducing risk across the whole population would be hypothesized to achieve a greater impact on incidence.

Our findings are consistent with a recent paper from Kivimaki et al.,^23^ which found that four dementia risk scores (including CAIDE 1, CAIDE 2, and ANU–ADRI) had failure rates (incident cases arising from normal‐risk group) of 84% to 91% when a cut‐off that minimized false positives (non‐cases in high‐risk group) was applied. This was no better than if age alone were used as a risk score. These analyses were done in UK Biobank, a very large but non‐representative cohort with a baseline response rate of only 5% and a follow‐up of only 10 years.^24^ The consistency with the results of our analyses in a cohort with much greater population representativeness and with almost two decades longer follow‐up therefore adds internal and external validity to these findings.

Our findings demonstrate that most future dementia cases would be missed by a targeted high‐risk approach. Importantly, even those included by such a targeted intervention would not be guaranteed to benefit. By definition, individual‐level interventions do not change the social and commercial determinants of health but aim to assist individuals to lower their own risk in spite of these factors, and as a result most are unlikely to benefit.^7^, ^25^ Moreover, individual‐level interventions (eg, advising people to reduce their salt intake) typically require more agency (the resources required to benefit from the intervention) on the part of the individual than population‐level interventions (eg, reformulation of products to reduce salt in the food supply^14^). Agency is not evenly distributed across societies but instead follows a socioeconomic gradient.^25^, ^26^ Therefore, individual‐level approaches typically widen health inequalities, whereas population‐level interventions can narrow them.^27^ Further, the association between higher risk scores and mortality speaks to the need for health initiatives to move beyond individual‐level disease‐specific siloes to reflect the interconnected nature of lifecourse risk factors with chronic disease, multimorbidity, and frailty in the older population.

### 4.3 Limitations

Data on dementia diagnoses came from linkage with secondary‐care inpatient data (ie, hospital admissions), Mental Health Trusts (ie, memory clinics), and mortality data, but not from primary‐care records. A small substudy conducted in EPIC‐Norfolk, which involved manually reviewing primary‐care records, suggested that up to 40% of total cases may be captured only by primary‐care data.^17^ Further work suggests that this is likely to represent a delay in reporting through to secondary care data sources, rather than a systematic bias for any specific group of patients, with socioeconomic status noted to be similar between dementia cases ascertained from different data sources.^17^ Thus, it is expected that our study identified the majority of incident cases and that missed cases (because they only appeared in primary‐care records) did not introduce a directional bias in our analyses.

Screening programs are sometimes designed to re‐screen “negative” individuals after a specified amount of time. Though associated with increased cost and resource requirements, this is intended to enable the identification of those who move from normal risk to high risk at some point after the initial screening. It is therefore possible that our approach of applying the risk scores only at baseline underestimated the number of future cases that would have been correctly classified as high risk by a screening program with repeated assessments of risk. Our selection of top quintiles and top deciles of risk to represent high‐risk groups were based upon how large a group it might be feasible to include in a hypothesized intervention, rather than an a priori defined score that corresponded to a specific level of risk.

Not all risk score variables were captured in EPIC‐Norfolk in the exact same way as in the original risk score derivation and/or validation cohorts. For the most part these discrepancies were trivial (Table S1). One variable (history of traumatic brain injury, ANU–ADRI) was missing altogether from EPIC‐Norfolk and was excluded from the 13‐item risk score. This was also the case in some of the external validation analyses conducted for ANU–ADRI by the risk score creators.^22^ For cognitive activity (LIBRA and ANU–ADRI) and social activity (ANU–ADRI) we tested the effect of using occupational social class and marital status as proxy variables in sensitivity analyses, and results were consistent with the primary analysis (Table S9). We did not perform multiple imputation to account for missing data. For 14 of the 17 indicators that constituted the risk scores, missing data were sparse (<3% of individuals missing data), and sensitivity analyses excluding specific variables with outlying amounts of missing data and using percentage risk scores did not meaningfully change the findings.

### 4.4 Conclusion

In this population‐based sample with 29 years of follow‐up, we found empirical evidence that Rose's prevention paradox did apply to dementia. Most future cases of dementia will arise from people considered at normal risk, not those at high risk. In addition, most people at high risk did not develop dementia over the next 29 years. Individual‐level dementia risk reduction programs that target high‐risk groups only are therefore unlikely to meaningfully reduce the incidence of dementia at the population level, and risk reduction across the whole population may be required.

## AUTHOR CONTRIBUTIONS

Sebastian Walsh, Lindsay Wallace, Oliver Mytton, Louise Lafortune, and Carol Brayne designed the study. Sebastian Walsh carried out the analysis, and Robert Luben checked the coding. Sebastian Walsh drafted the manuscript. All authors (Sebastian Walsh, Lindsay Wallace, Richard Merrick, Robert Luben, Shabina Hayat, Oliver Mytton, Louise Lafortune, Carol Brayne) reviewed and commented on the final manuscript.

## CONFLICT OF INTEREST STATEMENT

The authors declare no competing interests. Author disclosures are available in the supporting information.

## CONSENT STATEMENT

All human subjects provided informed consent.

## OPEN ACCESS

For the purpose of open access, the author has applied a Creative Commons Attribution (CC BY) license to any author accepted manuscript version arising from this submission.

## Supporting information

Supporting information

Supporting information

Supporting information

## Data Availability

EPIC‐Norfolk data are available upon request from the management team (https://www.epic‐norfolk.org.uk/for‐researchers/data‐sharing/data‐requests/). The code for our analysis is available from the authors upon request.

## References

[1] Prince M , Knapp M , Guerchet M , et al. 2014. Dementia UK: Update Second Edition.

[2] Wittenberg R , Knapp M , Hu B , et al. The costs of dementia in England. Int J Geriatr Psychiatry. 2019;34(7):1095‐1103. doi:10.1002/gps.5113 30950106 PMC6618309

[3] Walsh S , Merrick R , Richard E , Nurock S , Brayne C . Lecanemab for Alzheimer's disease. BMJ. 2022;379:o3010. Published online December 19, 2022:o3010. doi:10.1136/bmj.o3010 36535691

[4] Matthews FE , Arthur A , Barnes LE , et al. A two‐decade comparison of prevalence of dementia in individuals aged 65 years and older from three geographical areas of England: results of the Cognitive Function and Ageing Study I and II. Lancet. 2013;382(9902):1405‐1412. doi:10.1016/S0140-6736(13)61570-6 23871492 PMC3906607

[5] Livingston G , Huntley J , Sommerlad A , et al. Dementia prevention, intervention, and care: 2020 report of the Lancet Commission. Lancet. 2020;396(10248):413‐446. doi:10.1016/S0140-6736(20)30367-6 32738937 PMC7392084

[6] Walsh S , Govia I , Peters R , et al. What would a population‐level approach to dementia risk reduction look like, and how would it work? Alzheimer's & Dementia. 2023;19(7):3203‐3209. doi:10.1002/alz.12985 36791256

[7] Walsh S , Merrick R , Brayne C . The relevance of social and commercial determinants for neurological health. Lancet Neurol. 2022;21(12):1151‐1160. doi:10.1016/S1474-4422(22)00428-8 36402161

[8] Rose GA , Khaw KT , Marmot M . Rose's Strategy of Preventive Medicine: The Complete Original Text. Oxford University Press; 2008.

[9] Walsh S , Wallace L , Kuhn I , et al. Are population‐level approaches to dementia risk reduction under‐researched? A rapid review of the dementia prevention literature. J Prev Alzheimers Dis. 2024;11(1):241‐248. Published online 2023. doi:10.14283/jpad.2023.57 38230737

[10] Hou XH , Feng L , Zhang C , Cao XP , Tan L , Yu JT . Models for predicting risk of dementia: a systematic review. J Neurol Neurosurg Psychiatry. 2019;90(4):373‐379. 29954871 10.1136/jnnp-2018-318212

[11] Hing Tang EY , Robinson L , Maree Stephan BC , Dementia risk assessment tools: an update. Published online 2017. 10.2217/nmt-2017-0031 29160146

[12] Anstey KJ , Zheng L , Peters R , et al. Dementia risk scores and their role in the implementation of risk reduction guidelines. Front Neurol. 2022;12. doi:10.3389/fneur.2021.765454 PMC8764151 35058873

[13] Vonk JMJ , Greving JP , Gudnason V , Launer LJ , Geerlings MI . Dementia risk in the general population: large‐scale external validation of prediction models in the AGES‐Reykjavik study. Eur J Epidemiol. 2021;36(10). doi:10.1007/s10654-021-00785-x PMC8542560 34308533

[14] Walsh S , Wallace L , Kuhn I , et al. Population‐level interventions for the primary prevention of dementia: a complex evidence review. EClinicalMedicine. 2024;70:102538. doi:10.1016/j.eclinm.2024.102538 38495526 PMC10940136

[15] Walsh S , Roscoe H , Mathie E , Wallace L , Govia I , Brayne C . Exploring English policymakers’ attitudes towards dementia risk reduction: a qualitative study. Int J Geriatr Psychiatry. 2023;38(10). doi:10.1002/gps.6009 37794627

[16] Day N , Oakes S , Luben R , et al. EPIC‐Norfolk: study design and characteristics of the cohort. European Prospective Investigation of Cancer. Br J Cancer. 1999;80:95‐103. 10466767

[17] Hayat S , Luben R , Khaw KT , Wareham N , Brayne C . Evaluation of routinely collected records for dementia outcomes in UK: a prospective cohort study. BMJ Open. 2022;12(6). doi:10.1136/bmjopen-2022-060931 PMC9204445 35705339

[18] Luben R , Hayat S , Khawaja A , Wareham N , Pharoah PP , Khaw KT . Residential area deprivation and risk of subsequent hospital admission in a British population: the EPIC‐Norfolk cohort. BMJ Open. 2019;9(12). doi:10.1136/bmjopen-2019-031251 PMC6937051 31848162

[19] Kivipelto M , Ngandu T , Laatikainen T , Winblad B , Soininen H , Tuomilehto J . Risk score for the prediction of dementia risk in 20 years among middle aged people: a longitudinal, population‐based study. Lancet Neurol. 2006;5(9):735‐741. doi:10.1016/S1474-4422(06)70537-3 16914401

[20] Schiepers OJG , Köhler S , Deckers K , et al. Lifestyle for Brain Health (LIBRA): a new model for dementia prevention. Int J Geriatr Psychiatry. 2018;33(1):167‐175. doi:10.1002/gps.4700 28247500

[21] Verhaaren BFJ , Vernooij MW , Koudstaal PJ , et al. Alzheimer's disease genes and cognition in the nondemented general population. Biol Psychiatry. 2013;73(5). doi:10.1016/j.biopsych.2012.04.009 22592056

[22] Anstey KJ , Cherbuin N , Herath PM , et al. A self‐report risk index to predict occurrence of dementia in three independent cohorts of older adults: the ANU‐ADRI. PLoS One. 2014;9(1). doi:10.1371/journal.pone.0086141 PMC3900468 24465922

[23] Kivimäki M , Livingston G , Singh‐Manoux A , et al. Estimating dementia risk using multifactorial prediction models. JAMA Netw Open. 2023;6(6). doi:10.1001/jamanetworkopen.2023.18132 PMC10265307 37310738

[24] Brayne C , Moffitt TE . The limitations of large‐scale volunteer databases to address inequalities and global challenges in health and aging. Nat Aging. 2022;2(9):775‐783. doi:10.1038/s43587-022-00277-x 37118500 PMC10154032

[25] Marteau TM , Rutter H , Marmot M . Changing behaviour: an essential component of tackling health inequalities. bmj. 2021;372. 10.1136/bmj.n332 PMC7879795 33568384

[26] Adams J , Mytton O , White M , Monsivais P . Why are some population interventions for diet and obesity more equitable and effective than others? The role of individual agency. PLoS Med. 2016;13(4). doi:10.1371/journal.pmed.1001990 PMC4821622 27046234

[27] Walsh S , Wallace L , Mukadam N , et al. What is a population‐level approach to prevention, and how could we apply it to dementia risk reduction? Public Health. 2023;225:22‐27. doi:10.1016/j.puhe.2023.09.019 37918173

