# Supplementary material for: How many future dementia cases would be missed by a high‐risk screening program? A retrospective cohort study in a population‐based cohort
Source: Alzheimers Dement. 2024 Jul 18;20(9):6278–86. doi: 10.1002/alz.14113 (PMC11497639; doi:10.1002/alz.14113)
Supplement: Supplementary file 1 — Supporting information [file ALZ-20-6278-s002.docx]

# Supplementary Tables

| Supplementary Table S1 – Risk score coding approach | | |
| --- | --- | --- |
| Risk/Protective factor | Coding approach in derivation cohort | Coding approach in EPIC-Norfolk |
| **LIBRA Score**^1^ | | |
| High cognitive activity | Self-reported average number of hours/week reading or doing mental exercise (e.g. gaming)  Highest tertile = -3.2 | Excluded due to lack of data, sensitivity analysis to test proxy measure of occupational social class (managerial or professional = -3.2) |
| Mediterranean diet | Not available in derivation cohort, those with healthy diet would have scored = -1.7 | Coded according to Tong et al.^2^  Top tertile = -1.7 |
| Alcohol intake | Self-reported average units/week  >0, <14 = -1 | Self-reported average units/week >0, <14 = -1 |
| Coronary heart disease | Self-reported ‘currently suffering from heart disease or vascular disorders’ = 1  (Personal correspondence with LIBRA authors. Re: ‘CHD’, ideal is angina, ischaemic heart disease and myocardial infarction. But due to data availability they have sometimes included other conditions, e.g. atrial fibrillation, peripheral vascular disease etc. because they’ve been bundled in the data collection) | Self-reported history of angina, self-reported history of myocardial infarction, and linked secondary care data on ischaemic heart disease occurring during baseline data collection period = 1 |
| Physical inactivity | Self-reported ‘average number of hours up and about per day’  Lowest tertile = 1.1 | Those considered physically inactivity according to the analysis in EPIC-Norfolk by Luben et al.^3^ (lowest 32% of participants) = 1.1 |
| Renal dysfunction | Self-reported ‘currently suffering from renal disease’ = 1.1 | Linked secondary care data on renal disease occurring during baseline data collection period = 1.1 |
| Diabetes | Self-reported ‘currently suffering from diabetes’ = 1.3 | Self-reported diabetes or measured HBA1c >6.5% or linked secondary care data on diabetes occurring during baseline data collection period = 1.3 |
| High cholesterol | Self-reported high cholesterol, or reporting being on cholesterol-lowering medication = 1.4 | Self-reported high cholesterol, taking a statin, or measured total cholesterol >=6.5mmol/L (as per the LIBRA external validation paper in the CAIDE cohort)^4^ = 1.4 |
| Smoking | Currently smoking = 1.5 | Currently smoking = 1.5 |
| Obesity | Measured body mass index ≥30 = 1.6 | Measured body mass index ≥30, or self-reported if measured data not available = 1.6 |
| Hypertension | Measured blood pressure ≥140 (systolic) ≥90 (diastolic), or taking anti-hypertensives = 1.6 | Measured blood pressure ≥140 (systolic) ≥90 (diastolic), or self-reported hypertension = 1.6 |
| Depression | Depressive state measured by subscale of ‘Symptom Checklist90’  Top quartile = 2.1 | Self-reported medical diagnosis of depression, or taking an anti-depressant = 2.1 |
| **CAIDE Score 1**^5^ | | |
| Age | <47 = 0  47-53 = 3  >53 = 4 | Age at first questionnaire or first clinic assessment, whichever younger  <47=0, 47-53=3, >53=4 |
| Education | ≥10 years formal education = 0  7-9 years = 2  0-6 years = 3 | Years of formal education calculated by subtracting 4 from school leaving age. Additionally, include those who completed 10-12 years of schooling (i.e. left school at/before 16), but left without any qualifications, as score 2. |
| Sex | Male = 1 | Male =1 |
| SBP | Clinically measured  >140mmHg = 2 | ≥140mmHg systolic, ≥90mmHg diastolic, or self-reported hypertension =2 |
| BMI | Clinically measured  >30Kg/m^2^ = 2 | Clinically measured, or self-report if clinical measurement not available  ≥30 Kg/m^2^ =2 |
| Cholesterol | Clinically measured total cholesterol  >6.5mmol/L = 2 | Total cholesterol >=6.5mmol/L, self-reported hypercholesterolaemia, or statin use =2 |
| Physical inactivity | Not engaged in at least 2 physical activity sessions/week, at least 20-30 mins/session, causing sweating and breathlessness = 1 | Those considered physically inactivity according to the analysis in EPIC-Norfolk by Luben et al.^3^ (lowest 32% of participants) = 1.1 |
| **CAIDE Score 2**^5^ |  |  |
| Age | <47 = 0  47-53 = 3  >53 = 5 | Age at first questionnaire or first clinic assessment, whichever younger  <47=0, 47-53=3, >53=5 |
| Education | ≥10 years formal education = 0  7-9 years = 3  0-6 years = 4 | Years of formal education calculated by subtracting 4 from school leaving age. Additionally, include those who completed 10-12 years of schooling (i.e. left school at/before 16), but left without any qualifications, as score 3. |
| Sex | Male = 1 | Male =1 |
| SBP | Clinically measured  >140mmHg = 2 | ≥140mmHg systolic, ≥90mmHg diastolic, or self-reported hypertension =2 |
| BMI | Clinically measured  >30Kg/m^2^ = 2 | Clinically measured, or self-report if clinical measurement not available  ≥30 Kg/m^2^ =2 |
| Cholesterol | Clinically measured total cholesterol  >6.5mmol/L = 2 | Total cholesterol >=6.5mmol/L, self-reported hypercholesterolaemia, or statin use =2 |
| Physical inactivity | Not engaged in at least 2 physical activity sessions/week, at least 20-30 mins/session, causing sweating and breathlessness = 1 | Those considered physically inactivity according to the analysis in EPIC-Norfolk by Luben et al.^3^ (lowest 32% of participants) = 1.1 |
| APOE | ≥1 APOE-e4 allele = 2 | ≥1 APOE-e4 allele = 2 |
| **ANU-ADRI**^6,7^ | | |
| Age (males) | <65 = 0, ≥65, <70 = 1, ≥70, <75 = 12, ≥75, <80 = 18, ≥80, <85 = 26, ≥85, <90 = 33, ≥90 = 38 | Scoring as per left, using youngest age at first questionnaire or first clinical assessment |
| Age (females) | <65 = 0, ≥60, <70 = 5, ≥70, <75 = 14, ≥75, <80 = 21, ≥80, <85 = 29, ≥85, <90 = 35, ≥90 = 41 | Scoring as per left, using youngest age at first questionnaire or first clinical assessment |
| Education | Years of formal education  >11 = 0  8 – 11 = 3  <8 = 6 | Years of formal education calculated by subtracting 4 from school leaving age. Additionally, include those who completed 11-12 years of schooling (i.e. left school at/before 16), but left without any qualifications, as score 3. |
| BMI | BMI ≥25 = 2  BMI ≥30 = 5  N.B. only applied for <60s | Measured BMI, or self-report if measured BMI unavailable. ≥25=2, ≥30=5. Score all >=60s as 0 |
| Diabetes | No diabetes = 0  Diabetes (self-report, anti-diabetic meds, lab tests) = 3 | Self-reported diabetes, hospital recorded diabetes at baseline, HBA1c >6.5% (48mmol/mol) = 3 |
| Depression | CES-D ≥16 = 2 | Self-reported diagnosis of depression or anti-depressant use = 2 |
| Cholesterol | ≥6.2mmol/L = 3  <60s only | >=6.2 or self-report of high cholesterol or statin use = 3. Score all >=60s as 0 |
| Traumatic Brain Injury | Self-reported history of traumatic brain injury = 4 | Exclude due to lack of data |
| Smoking | Ever = 1  Current = 4 | 1 = Ever (not current)  4 = Current |
| Alcohol | Light-moderate = -3 | >0, <14 units/week = -3 |
| Social Engagement | 5 domains: marital status, size of social network, quality of social network, level of social activities, living arrangements. Scored 0, 1, 4, 6 depending on number of low scores | Exclude due to lack of data, but test marital status in sensitivity analysis, score 0 if married, otherwise 1 |
| Physical Activity | Calculated METs via the International Physical Activity Questionnaire, which measures frequency, duration, and intensity. Split cohort into tertiles. Light = 0, moderate = -2, high = -3 | Use variable from analysis in EPIC-Norfolk by Luben et al.^3^. Moderately active = -2, Active = -3 |
| Cognitive Activity | RUSH cohort questionnaire to assess leisure-time cognitive activities. Low = 0, moderate = -6, high = -7 | Excluded due to lack of data, sensitivity analysis to test proxy measure of occupational social class. Managerial = -6, professional = -7 |
| Fish Intake | ≥0.25, <2 portions/week = -3  ≥2, <4 portions/week = -4  ≥4 portions/week = -5 | Combined consumption of oily and white fish, ≥0.25 <2 = -3, ≥2, <4 =-4, >4 = -5 |
| Pesticide Exposure | Ever = 2 | Self-report, ever worked in arable farming using pesticides or herbicides for at least one year |

| Supplementary Table S2 – Performance of the risk prediction scores in EPIC-Norfolk by age group | | | | | | |
| --- | --- | --- | --- | --- | --- | --- |
| **Risk score** | **High-risk threshold** | | **Cut-point value** | **Percentage of cohort high-risk at baseline**  % (95% CI) | **Percentage of cases arising from high-risk group**  % (95% CI) | **Dementia cases/ 1000 high-risk**  n (95% CI) |
| **50-59 year olds (total incidence rate 6.6%)** | | | | | | |
| CAIDE 1 | Top 10% | | ≥ 11 | 5.3 (4.8, 5.8) | 7.4 (5.3, 9.5) | 92 (66, 117) |
|  | Top 20% | | ≥ 10 | 11.5 (10.8, 12.1) | 15.1 (12.3, 18.0) | 87 (70, 104) |
|  |  | |  |  |  |  |
| CAIDE 2 | Top 10% | | ≥ 12 | 9.6 (8.9, 10.3) | 19.2 (15.5, 22.9) | 129 (103, 154) |
|  | Top 20% | | ≥ 11 | 17.5 (16.6, 18.4) | 30.4 (26.1, 34.7) | 112 (94, 130) |
|  |  | |  |  |  |  |
| LIBRA | Top 10% | | ≥ 3.9 | 9.8 (9.1, 10.4) | 11.4 (8.5, 14.3) | 73 (55, 92) |
|  | Top 20% | | ≥ 3.0 | 19.5 (18.6, 20.4) | 21.7 (18.0, 25.4) | 70 (57, 83) |
|  |  | |  |  |  |  |
| ANU-ADRI | Top 10% | | ≥ 6 | 8.0 (7.4, 8.6) | 8.9 (6.4, 11.4) | 72 (52, 92) |
|  | Top 20% | | ≥ 4 | 14.8 (14.0, 15.6) | 16.8 (13.6, 20.1) | 73 (58, 88) |
| APOE e4 carriership | Male | |  | 29.3 (27.7, 30.9) | 50.9 (44.3, 57.6) | 121 (100, 142) |
|  | Female | |  | 28.4 (27.0, 29.9) | 57.2 (50.7, 63.7) | 122 (102, 142) |
| **60-69 year olds (total incidence rate 20.1%)** | | | | | | |
| CAIDE 1 | Top 10% | | ≥ 12 | 5.5 (5.0, 6.0) | 5.2 (4.2, 6.2) | 197 (162, 232) |
|  | Top 20% | | ≥ 11 | 13.3 (12.6, 14.0) | 14.1 (12.5, 15.7) | 221 (197, 244) |
|  |  | |  |  |  |  |
| CAIDE 2 | Top 10% | | ≥ 14 | 6.1 (5.6, 6.7) | 8.4 (6.9, 9.9) | 286 (241, 330) |
|  | Top 20% | | ≥ 13 | 13.2 (12.4, 14.0) | 15.8 (13.9, 17.7) | 249 (221 278) |
|  |  | |  |  |  |  |
| LIBRA | Top 10% | | ≥ 4.3 | 9.9 (9.2, 10.6) | 9.9 (8.4, 11.4) | 214 (183, 244) |
|  | Top 20% | | ≥ 3.2 | 19.9 (19.0, 20.8) | 21.3 (19.3, 23.4) | 228 (206, 250) |
|  |  | |  |  |  |  |
| ANU-ADRI | Top 10% | | ≥ 5 | 7.5 (7.0, 8.1) | 8.2 (6.8, 9.5) | 226 (192, 260) |
|  | Top 20% | | ≥ 3 | 15.0 (14.2, 15.8) | 16.3 (14.5, 18.1) | 227 (203, 251) |
| APOE e4 carriership | Male | |  | 27.2 (25.6, 28.7) | 41.0 (37.0, 45.1) | 272 (242, 301) |
|  | Female |  | | 28.2 (26.7, 29.8) | 42.1 (38.6, 45.5) | 351 (320, 381) |

| Supplementary table S3 – Association between high-risk group status and age at diagnosis from linear regression models adjusted for baseline age | | | |
| --- | --- | --- | --- |
| High-risk group | Coefficient | 95% confidence interval | P value |
| CAIDE 1 top decile | 0.03 | -0.42, 0.49 | 0.885 |
| CAIDE 1 top quintile | -0.12 | -0.47. 0.24 | 0.521 |
|  |  |  |  |
| CAIDE 2 top decile | -0.89 | -1.36, -0.41 | <0.001 |
| CAIDE 2 top quintile | -0.64 | -1.03, -0.25 | 0.001 |
|  |  |  |  |
| LIBRA top decile | -0.69 | -1.25, -0.13 | 0.016 |
| LIBRA top quintile | -0.63 | -1.02, -0.24 | 0.002 |
|  |  |  |  |
| ANU-ADRI top decile | -1.44 | -1.94, -0.94 | <0.001 |
| ANU-ADRI top quintile | -1.59 | -2.03, -1.15 | <0.001 |
|  |  |  |  |
| APOE e4 carriership (male)* | -2.14 | -2.90, -1.38 | <0.001 |
| APOE e4 carriership (female)* | -2.58 | -3.23, -1.94 | <0.001 |
| All models adjusted for baseline age, because each high-risk group statistically significantly older at baseline than corresponding normal-risk group. With the exception of APOE e4, as there was no difference in baseline age between carriers and non-carriers, this model is not adjusted for baseline age. | | | |

| Supplementary Table S4 – Performance of the risk prediction scores in EPIC-Norfolk by diagnostic subgroup | | | | | |
| --- | --- | --- | --- | --- | --- |
| **Risk score** | **High-risk threshold** | **Cut-point value** | **Percentage of cohort high-risk at baseline**  % (95% CI) | **Percentage of cases arising from high-risk group**  % (95% CI) | **Dementia cases/ 1000 high-risk**  N (95% CI) |
| **Alzheimer’s disease (total incidence rate 4.5%)** | | | | | |
| CAIDE 1 | Top 10% | ≥ 11 | 8.6 (8.3, 8.9) | 11.8 (10.1, 13.6) | 61 (52, 71) |
|  | Top 20% | ≥ 10 | 16.3 (15.9, 16.8) | 22.6 (20.3, 24.8) | 62 (55, 69) |
|  |  |  |  |  |  |
| CAIDE 2 | Top 10% | ≥ 13 | 8.4 (8.0, 8.8) | 14.8 (12.6, 17.0) | 81 (69, 94) |
|  | Top 20% | ≥ 12 | 14.9 (14.4, 15.4) | 26.5 (23.8, 29.3) | 82 (73, 92) |
|  |  |  |  |  |  |
| LIBRA | Top 10% | ≥ 4.2 | 8.3 (7.9, 8.6) | 8.7 (7.0, 10.3) | 48 (39, 58) |
|  | Top 20% | ≥ 3.1 | 19.0 (18.5, 19.5) | 21.1 (18.7, 23.6) | 51 (45, 58) |
|  |  |  |  |  |  |
| ANU-ADRI | Top 10% | ≥ 11 | 8.8 (8.5, 9.2) | 14.5 (12.4, 16.5) | 75 (64, 86) |
|  | Top 20% | ≥ 6 | 19.8 (19.3, 20.3) | 30.0 (27.3, 32.6) | 69 (62, 76) |
| APOE e4 carriership | Male |  | 28.3 (27.4, 29.2) | 50.6 (45.4, 55.7) | 66 (56, 75) |
|  | Female |  | 28.6 (27.8, 29.5) | 51.2 (47.3, 55.2) | 98 (87, 108) |
| **Vascular dementia (total incidence rate 3.1%)** | | | | | |
| CAIDE 1 | Top 10% | ≥ 11 | 8.6 (8.3, 8.9) | 15.1 (12.8, 17.4) | 55 (46, 63) |
|  | Top 20% | ≥ 10 | 16.3 (15.9, 16.8) | 28.0 (25.1, 30.9) | 53 (47, 60) |
|  |  |  |  |  |  |
| CAIDE 2 | Top 10% | ≥ 13 | 8.4 (8.0, 8.8) | 18.4 (15.5, 21.3) | 72 (60, 84) |
|  | Top 20% | ≥ 12 | 14.9 (14.4, 15.4) | 30.0 (26.6, 33.5) | 66 (57, 75) |
|  |  |  |  |  |  |
| LIBRA | Top 10% | ≥ 4.2 | 8.3 (7.9, 8.6) | 10.9 (8.6, 13.1) | 40 (32, 49) |
|  | Top 20% | ≥ 3.1 | 19.0 (18.5, 19.5) | 26.0 (22.8, 29.3) | 42 (36, 48) |
|  |  |  |  |  |  |
| ANU-ADRI | Top 10% | ≥ 11 | 8.8 (8.5, 9.2) | 14.8 (12.3, 17.3) | 53 (44, 62) |
|  | Top 20% | ≥ 6 | 19.8 (19.3, 20.3) | 30.4 (27.2, 33.7) | 49 (43, 55) |
| APOE e4 carriership | Male |  | 28.3 (27.4, 29.2) | 41.3 (36.1, 46.5) | 51 (42, 59) |
|  | Female |  | 28.7 (27.8, 29.5) | 41.7 (36.5, 46.9) | 45 (38, 52) |
| **Other dementia (total incidence rate 8.9%)** | | | | | |
| CAIDE 1 | Top 10% | ≥ 11 | 8.6 (8.3, 8.9) | 13.0 (11.7, 14.3) | 132 (119, 146) |
|  | Top 20% | ≥ 10 | 16.3 (15.9, 16.8) | 24.0 (22.3, 25.6) | 129 (120, 139) |
|  |  |  |  |  |  |
| CAIDE 2 | Top 10% | ≥ 13 | 8.4 (8.0, 8.8) | 15.8 (14.1, 17.4) | 164 (147, 181) |
|  | Top 20% | ≥ 12 | 14.9 (14.4, 15.4) | 27.6 (25.5, 29.6) | 162 (149, 175) |
|  |  |  |  |  |  |
| LIBRA | Top 10% | ≥ 4.2 | 8.3 (7.9, 8.6) | 10.2 (8.9, 11.5) | 109 (95, 123) |
|  | Top 20% | ≥ 3.1 | 19.0 (18.5, 19.5) | 24.7 (22.8, 26.6) | 115 (105, 124) |
|  |  |  |  |  |  |
| ANU-ADRI | Top 10% | ≥ 11 | 8.8 (8.5, 9.2) | 17.7 (16.1, 19.3) | 177 (161, 193) |
|  | Top 20% | ≥ 6 | 19.8 (19.3, 20.3) | 34.7 (32.8, 36.7) | 155 (145, 166) |
| APOE e4 carriership | Male |  | 28.3 (27.4, 29.2) | 43.8 (40.3, 47.3) | 120 (108, 132) |
|  | Female |  | 28.6 (27.8, 29.5) | 42.6 (39.7, 45.6) | 143 (131, 155) |

| Supplementary Table S5 – Missing data analysis | | | | | |
| --- | --- | --- | --- | --- | --- |
| **Risk Score** | **Missing data** | **Age (years)** | **Sex** | **SES (Townsend Index)** | **Incident dementia** |
|  | n, % | dif (95% CI), p val | women, men, p val | dif (95% CI), p val | cases, non-cases, p val |
| CAIDE 1 | 914 (3.0%) | 2.60 (1.97, 3.22), p<0.001 | 3.49%, 2.40%, p<0.001 | 0.66 (0.51, 0.80), p<0.001 | 3.29%, 2.95%, p=0.245 |
|  |  |  |  |  |  |
| CAIDE 2 | 9,415 (30.9%) | 0.78 (0.56, 1.01), p<0.001 | 32.9%, 28.6%, p<0.001 | 0.34 (0.28, 0.39), p<0.001 | 30.7%, 30.9%, p=0.813 |
|  |  |  |  |  |  |
| LIBRA | 7,157 (23.5%) | 2.20 (1.96, 2.45), p<0.001 | 22.5%, 24.8%, p<0.001 | 0.42 (0.36, 0.48), p<0.001 | 23.7%, 23.4%, p=0.724 |
|  |  |  |  |  |  |
| ANU-ADRI | 5,582 (18.3%) | 0.90 (0.62, 1.17), p<0.001 | 18.9%, 17.6%, p=0.005 | 0.43 (0.37, 0.50), p<0.001 | 18.3%, 18.3%, p=0.972 |
|  |  |  |  |  |  |
| APOE e4 | 9,396 (30.9%) | 0.78 (0.55, 1.01), p<0.001 | 32.8%, 28.5%, p<0.001 | 0.33 (0.28, 0.39), p<0.001 | 30.7%, 30.9%, p=0.853 |
| SES = Socioeconomic status. Dif = Difference. CI = Confidence interval. p val = p value  Missing data by age and SES compared by t test, comparing the average for those with missing data to those not missing data (i.e. for CAIDE 1, those missing data were on average 2.6 years older). Townsend index ranged between -6.73 to 6.98, with a higher Townsend index score indicating a more deprived area. Missing data by sex and incident dementia compared using chi-squared tests (reported as the % of women who had missing data etc.). | | | | | |

| Supplementary Table S6 – Sensitivity analysis removing individual variables with outlying amounts of missing data | | | | | | | | | | | | |
| --- | --- | --- | --- | --- | --- | --- | --- | --- | --- | --- | --- | --- |
| **Missing data by risk score (those removed in sensitivity analysis in bold)** | | | | | | | | | | | | |
| CAIDE | Age | Education | Sex | Hypertension | Obesity | Cholesterol | Physical Activity | |  |  |  |  |
|  | 15 | 33 | 0 | 17 | 894 | 0 | 1 | |  |  |  |  |
|  |  |  |  |  |  |  |  | |  |  |  |  |
| LIBRA | **Diet** | Alcohol | CHD | Hypertension | Renal | Cholesterol | Physical Activity | | Smoking | Obesity | Diabetes | Depression |
|  | **6745** | 393 | 0 | 17 | 0 | 0 | 1 | | 286 | 894 | 0 | 0 |
|  |  |  |  |  |  |  |  | |  |  |  |  |
| ANU-ADRI | Age | Education | Obesity | Diabetes | Pesticide | Cholesterol | Physical Activity | | Alcohol | Smoking | **Fish Intake** | Depression |
|  | 15 | 0 | 369 | 0 | 0 | 0 | 286 | | 393 | 286 | **5,128** | 0 |
| **Risk Score** | | **High-risk threshold** | | **Cut-point value** | **Percentage of cohort high-risk at baseline**  % (95% CI) | | | **Percentage of cases arising from high-risk group**  % (95% CI) | | | **Dementia cases/**  **1000 at high-risk**  n (95% CI) | |
| LIBRA (no diet) | | Top 10% | | ≥ 2.7 | 9.3% (9.0, 9.7) | | | 10.8% (9.8, 11.8) | | | 143 (130, 157) | |
|  | | Top 20% | | ≥ 1.6 | 19.3% (18.8, 19.8) | | | 22.9% (21.5, 24.2) | | | 146 (137, 156) | |
|  | |  | |  |  | | |  | | |  | |
| ANU-ADRI (no fish) | | Top 10% | | ≥ 9 | 9.9% (9.5, 10.2) | | | 19.1% (17.8, 20.3) | | | 241 (225, 256) | |
|  | | Top 20% | | ≥ 5 | 17.5% (17.0, 17.9) | | | 30.7% (29.2, 32.2) | | | 220 (208, 231) | |
| CHD = Coronary Heart Disease | | | | | | | | | | | | |

| Supplementary Table S7 – Sensitivity analysis using % risk scores with all available data | | | | | |
| --- | --- | --- | --- | --- | --- |
| **Risk score** | **High-risk threshold** | **Percentage of cohort**  **high-risk at baseline**  % (95% CI) | **Percentage of cases arising**  **from high-risk group**  % (95% CI) | | **Dementia cases/ 1000 high-risk***  n (95% CI) |
| CAIDE 1 | Top 10% | 8.7% (8.4, 9.0) | 12.9% (11.9, 14.0) | | 186 (171, 201) |
|  | Top 20% | 16.5% (16.1, 17.0) | 24.0% (22.7, 25.4) | | 182 (171, 192) |
|  |  |  |  | |  |
| CAIDE 2 | Top 10% | 8.7% (8.4, 9.0) | 14.8% (13.6, 15.9) | | 212 (196, 228) |
|  | Top 20% | 18.6% (18.1, 19.0) | 30.0% (28.6, 31.5) | | 202 (192, 213) |
|  |  |  |  | |  |
| LIBRA | Top 10% | 9.2% (8.9, 9.5) | 10.4% (9.5, 11.4) | | 142 (129, 155) |
|  | Top 20% | 18.0% (17.5, 18.4) | 21.5% (20.2, 22.8) | | 150 (140, 159) |
|  |  |  |  | |  |
| ANU-ADRI | Top 10% | 9.6% (9.3, 9.9) | 17.0% (15.8, 18.2) | | 222 (207, 237) |
|  | Top 20% | 19.2% (18.7, 19.6) | 32.7% (31.2, 34.2) | | 214 (203, 224) |
|  |  |  |  | |  |
| **Risk score** | **Mean % in incident dementia cases** | | **Mean % in non-cases** | **Absolute Difference** | |
| CAIDE 1 | 52.7% | | 43.9% | 8.8% | |
|  |  | |  |  | |
| CAIDE 2 | 54.8% | | 43.9% | 10.9% | |
|  |  | |  |  | |
| LIBRA | 28.3% | | 25.5% | 2.8% | |
|  |  | |  |  | |
| ANU-ADRI | 20.8% | | 16.8% | 4.0% | |

| Supplementary Table S8 – Sensitivity analysis removing incident cases in first 10 years of follow-up | | | | |
| --- | --- | --- | --- | --- |
| **Risk score** | **High-risk threshold** | **Percentage of cohort**  **high-risk at baseline**  % (95% CI) | **Percentage of cases arising**  **from high-risk group**  % (95% CI) | **Dementia cases/ 1000 high-risk***  n (95% CI) |
| CAIDE 1 | Top 10% | 8.6% (8.2, 8.9) | 12.6% (11.5, 13.7) | 173 (159, 188) |
|  | Top 20% | 16.2% (15.8, 16.6) | 23.5% (22.1, 24.9) | 170 (160, 181) |
|  |  |  |  |  |
| CAIDE 2 | Top 10% | 8.3% (7.9, 8.6) | 15.1% (13.7, 16.5) | 217 (198, 237) |
|  | Top 20% | 14.7% (14.2, 15.2) | 26.2% (24.5, 28.0) | 212 (198, 227) |
|  |  |  |  |  |
| LIBRA | Top 10% | 8.2% (7.9, 8.6) | 9.6% (8.5, 10.7) | 138 (123, 154) |
|  | Top 20% | 18.9% (18.4, 19.4) | 24.0% (22.4, 25.6) | 150 (140, 161) |
|  |  |  |  |  |
| ANU-ADRI | Top 10% | 8.6% (8.2, 8.9) | 14.5% (13.3, 15.8) | 201 (184, 218) |
|  | Top 20% | 19.4% (18.9, 19.9) | 30.0 (28.3, 31.7) | 183 (172, 194) |
|  |  |  |  |  |
| APOE e4 Carriership | Male | 28.1% (27.2, 29.0) | 43.3% (40.3, 46.3) | 162 (148, 176) |
|  | Female | 28.3% (27.5, 29.2) | 43.1% (40.6, 45.6) | 200 (186, 214) |
|  | | | | |

| Supplementary Table S9 – Sensitivity analysis including proxy variables of occupational social class for cognitive activity (LIBRA, ANU-ADRI), and marital status for social engagement (ANU-ADRI) | | | | |
| --- | --- | --- | --- | --- |
| **Risk score** | **High-risk threshold** | **Percentage of cohort**  **high-risk at baseline**  % (95% CI) | **Percentage of cases arising**  **from high-risk group**  % (95% CI) | **Dementia cases/ 1000 high-risk***  n (95% CI) |
| LIBRA | Top 10% | 10.0% (9.6, 10.4) | 13.5% (12.2, 14.8) | 167 (151, 182) |
|  | Top 20% | 19.6% (19.0, 20.1) | 24.8 (23.2, 26.4) | 156 (145, 167) |
|  |  |  |  |  |
| ANU-ADRI | Top 10% | 9.9% (9.5, 10.2) | 18.4% (17.0, 19.8) | 231 (214, 248) |
|  | Top 20% | 17.6% (17.1, 18.1) | 28.6% (27.0, 30.3) | 201 (189, 213) |
|  | | | | |

## References

1. Schiepers OJG, Köhler S, Deckers K, et al. Lifestyle for Brain Health (LIBRA): a new model for dementia prevention. *Int J Geriatr Psychiatry*. 2018;33(1):167-175. doi:10.1002/gps.4700

2. Tong TYN, Wareham NJ, Khaw KT, Imamura F, Forouhi NG. Prospective association of the Mediterranean diet with cardiovascular disease incidence and mortality and its population impact in a non-Mediterranean population: The EPIC-Norfolk study. *BMC Med*. 2016;14(1). doi:10.1186/s12916-016-0677-4

3. Luben R, Hayat S, Wareham N, Pharoah P, Khaw KT. Usual physical activity and subsequent hospital usage over 20 years in a general population: The EPIC-Norfolk cohort. *BMC Geriatr*. 2020;20(1). doi:10.1186/s12877-020-01573-0

4. Deckers K, Barbera M, Köhler S, et al. Long-term dementia risk prediction by the LIBRA score: A 30-year follow-up of the CAIDE study. *Int J Geriatr Psychiatry*. 2020;35(2). doi:10.1002/gps.5235

5. Kivipelto M, Ngandu T, Laatikainen T, Winblad B, Soininen H, Tuomilehto J. Risk score for the prediction of dementia risk in 20 years among middle aged people: a longitudinal, population-based study. *Lancet Neurol*. 2006;5(9):735-741. doi:https://doi.org/10.1016/S1474-4422(06)70537-3

6. Anstey KJ, Cherbuin N, Herath PM, et al. A self-report risk index to predict occurrence of dementia in three independent cohorts of older adults: The ANU-ADRI. *PLoS One*. 2014;9(1). doi:10.1371/journal.pone.0086141

7. Anstey KJ, Cherbuin N, Herath PM. Development of a New Method for Assessing Global Risk of Alzheimer’s Disease for Use in Population Health Approaches to Prevention. *Prevention Science*. 2013;14(4):411-421. doi:10.1007/s11121-012-0313-2
